# Phéochromocytome surrénalien: difficultés diagnostiques et thérapeutiques

**DOI:** 10.11604/pamj.2015.22.135.7998

**Published:** 2015-10-13

**Authors:** Manel Jellouli, Tahar Gargah

**Affiliations:** 1 Service de Pédiatrie, Hôpital Charles Nicolle, Tunis, Tunisie

**Keywords:** Pheochromocytome, enfant, hypertension artérielle, Pheochromocytoma, infant, hypertension

## Image en medicine

Le phéochromocytome est une tumeur rare chez l'enfant, ses manifestations cliniques résultent d'une excrétion croissante de catécholamines. L'hypertension artérielle est le signe révélateur principal. HMS, garçon âgé de 13 ans est hospitalisé pour hypertension artérielle sévère. Il se plaignait depuis plusieurs mois de céphalées, palpitations et d'accès de sueurs. L'examen physique a trouvé un enfant eutrophique, hypertendu (200/130 mm Hg) et tachycarde (135 pulsations par minute). L'examen cardiovasculaire était normal. L’électrocardiographe et l’échographie cardiaque était normaux. Il existait une rétinopathie hypertensive stade II au fond d’œil. La glycémie à jeun est à 6,5 mmol/litre; le taux des VMA à la limite supérieure de la normale (8mg/l). L’échographie abdominale a mis objectivée une masse hétérogène de 44 mm de grand axe en rapport avec la surrénale gauche. Au scanner, cette masse semble appendue à la queue du pancréas, donnée réfutée par l’échographie endoscopique (A). L'IRM abdominale a confirmé l'origine surrénalienne de la masse en montrant une formation ovalaire de 40×60 mm en hyposignal T 1 et hyper signal T 2, refoulant le rein gauche (B). Le patient fut opéré sous anesthésie générale après un équilibre tensionnel parfait. Le temps peropératoire a été marqué par la survenue d'un collapsus, suivi d'asystolie récupérée après 3 minutes de réanimation avec un état hémodynamique stable au cours et après l'intervention. Le phéochromocytome a été confirmé par l’étude histologique de la pièce opératoire. L'enfant âgé actuellement de 15 ans se porte bien avec des contrôles cliniques et échographiques satisfaisants.

**Figure 1:**
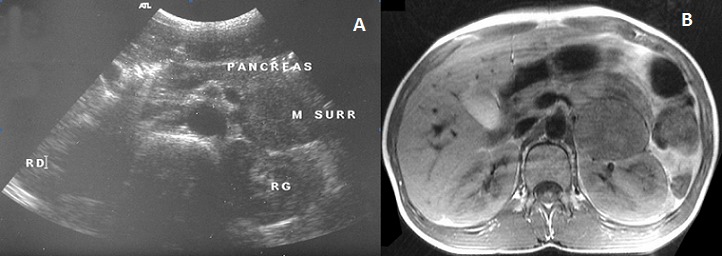
(A) échographie endoscopique montrant une masse surrénalienne gauche; (B) IRM abdominale: masse surrénalienne gauche, bien circonscrite et refoulant le rein en arrière

